# Imported malaria in Finland 2003-2011: prospective nationwide data with rechecked background information

**DOI:** 10.1186/1475-2875-12-93

**Published:** 2013-03-14

**Authors:** Heli Siikamäki, Pia Kivelä, Outi Lyytikäinen, Anu Kantele

**Affiliations:** 1 Department of Medicine, Division of Infectious Diseases, Helsinki University Central Hospital, Aurora Hospital, Building 5, 3rd floor, PO Box 348, Helsinki FI-00029, HUS, Finland; 2 National Institute for Health and Welfare, PO Box 30, Helsinki FI-00271, Finland; 3 University of Helsinki, Institute of Clinical Medicine, University of Helsinki, PO Box 20, Helsinki FIN-00014, Finland; 4 Department of Bacteriology and Immunology, University of Helsinki, Haartman Institute, University of Helsinki, PO Box 21, Helsinki FIN-00014, Finland

**Keywords:** Malaria, Imported malaria, Europe, Pre-travel advice, Visiting friends and relatives, VFR, Chemoprophylaxis, Mefloquine, Atovaquone/proguanil, Doxycycline

## Abstract

**Background:**

Although described in several reports, imported malaria in Europe has not been surveyed nationwide with overall coverage of patients and individually rechecked background information. *Plasmodium falciparum* infections have been reported despite regularly taken appropriate chemoprophylaxis, yet the reliability of such questionnaire-based retrospective data has been questioned. This was the starting-point for conducting a prospective nationwide survey of imported malaria where compliance data was double-checked.

**Methods:**

Data was collected on all cases of imported malaria confirmed and recorded by the reference laboratory of Finland (population 5.4 million) from 2003 to 2011, and these were compared with those reported to the National Infectious Disease Register (NIDR). Background information was gathered by detailed questionnaires sent to the clinicians upon diagnosis; missing data were enquired by telephone of clinician or patient. Special attention was paid to compliance with chemoprophylaxis: self-reported use of anti-malarials was rechecked for all cases of *P. falciparum*.

**Results:**

A total of 265 malaria cases (average annual incidence rate 0.5/100,000 population) had been recorded by the reference laboratory, all of them also reported to NIDR: 54% were born in malaria-endemic countries; 86% were currently living in non-endemic regions. Malaria was mainly (81%) contracted in sub-Saharan Africa. *Plasmodium falciparum* proved to be the most common species (72%). Immigrants constituted the largest group of travellers (44%). Pre-travel advice was received by 20% of those born in endemic regions and 81% of those from non-endemic regions. Of those with *P. falciparum*, 4% reported regular use of appropriate chemoprophylaxis (mefloquine or atovaquone/proguanil or doxycycline for regions with chloroquine-resistant and atovaquone/proguanil or doxycycline for regions with mefloquine-resistant *P. falciparum*); after individual rechecking, however, it was found that none of them had been fully compliant.

**Conclusions:**

Information on compliance with chemoprophylactic regimen cannot be relied on, and it should be rechecked if malaria is suspected. The results of the present study suggest that mefloquine, atovaquone/proguanil and doxycycline are effective as chemoprophylaxis against *P. falciparum* malaria, when taken conscientiously.

## Background

Malaria continues to be a major public health problem with over 200 million infections and approximately 655,000 deaths worldwide, 90% occurring in Africa [1]. The annual number of notified malaria cases imported into European countries has been 10,000–13,000 (2-3/100,000 population) [2,3] in previous years. In 2010, the World Health Organization (WHO) reported 6,244 cases of imported malaria in Europe [4], which is considered an underestimation [5]*.* Several studies deal with data solely from single European hospitals, cities, areas [6-9] and networks [10,11]. For most of them, and for a number of recent large reports containing national malaria surveillance data from Europe [12-16], merely limited background information has been collected or significant parts of pertinent data are missing. In all these surveys, the reliability of information collected by questionnaires has been highlighted as a problem. Despite appropriate chemoprophylaxis, cases of *Plasmodium falciparum* malaria have been reported. However, the use of anti-malarial drugs has been recorded retrospectively solely on the basis of questionnaires [13,15,17,18], without reconfirming regimen compliance.

The research group set out to prospectively collect and survey detailed data on all Finnish travellers with malaria, placing emphasis on compliance with chemoprophylaxis. The surveillance of imported malaria as well as travel trends and anti-malarial drug sales in Finland 1995-2008 has been discussed in an overview published earlier [19]. It showed that the number of trips to malaria-endemic areas increased during 2000-2008. Most infections were acquired in the same geographical regions as the immigrants’ country of birth. Another important finding was that travel advice is not getting through to immigrants visiting their friends and relatives (VFR) [19].

Malaria prophylaxis and treatment should be fairly uniform throughout Finland because of well-known national guidelines. The instructions for malaria prophylaxis are available for both health care professionals and the public in *Matkailijan terveysopas* (travellers’ health guide) [20] (first published in 1993) freely accessible and up-to-date on the National Institute for Health and Welfare (THL) website [21]. National guidelines for malaria diagnostics and treatment are presented in *Akuuttihoito-opas* (manual of emergency treatment) [22,23] continuously updated and available online for Finnish physicians.

Detailed background information was collected on imported malaria cases in Finland during 2003–2011 in order to identify risk groups, and to obtain information on both the use of chemoprophylaxis and adherence to the national guidelines. Of particular interest in the study was the question whether *P. falciparum* malaria can indeed be contracted in spite of appropriate chemoprophylaxis. To that end individual compliance data was rechecked from the clinician or the patient in all *P. falciparum* cases.

## Methods

### Surveillance of malaria in Finland

The National Infectious Disease Register (NIDR) was established, and malaria became a notifiable disease in Finland in 1995. Since then, all Finnish clinical microbiology laboratories conducting malaria diagnostics have reported positive tests to the NIDR. In addition, surveillance data have been collected from clinicians to the NIDR by a notification form, which covers such variables as age, sex, country of birth, diagnostic specimen date, and country of infection.

HUSLAB is the national reference laboratory where positive malaria slides from all Finnish laboratories are delivered for confirmation. In cases of unclear species identification, HUSLAB has, since the beginning of 2004, sent malaria smears over to the Swiss Tropical Institute for further affirmation with the polymerase chain reaction (PCR).

### Study design

For the present study, a case was defined as a person with *Plasmodium* infection confirmed by microscopy. Immediately after diagnosis HUSLAB sent detailed questionnaires to clinicians treating patients confirmed as malaria cases. The replies were forwarded to the author HS, who without delay rechecked and filled in any missing details from the patient records of those treated at the Helsinki University Central Hospital (HUCH), and consulted the clinicians, if the answers were found unclear or incomplete. If the clinicians were not able to provide the information needed, HS got in touch with the individual patients over the telephone. Data about compliance with prophylactic regimen was rechecked in all *P. falciparum* cases if the information was incomplete and, especially, if regular use of chemoprophylaxis was reported. The data obtained were consistently compared with the NIDR notifications, and used for the annual reports to the THL and the WHO.

The data comprised demographic variables (sex, age, country of birth, nationality, recent and previous countries of residence, date of immigration), accurate travel history (countries and areas visited plus dates and reason for travel), pre-travel advice received; use of chemoprophylaxis, description of symptoms, dates of onset, first contact with healthcare and presenting at hospital, diagnostic specimen date, malaria species, diagnostic method, date of commencing treatment, medication used, time and place of diagnosis, treatment and hospitalisation, as well as disease complications and outcome.

Symptoms and complications were enquired as an open question in the questionnaire, and were analysed only for patients treated at the HUCH, as their data could be obtained systematically from patient records throughout the study period.

### Ethics statement

The study protocol was approved by the Department of Internal Medicine of the Helsinki University Central Hospital. Background information was collected by the reference laboratory as part of its monitoring activity.

### Definitions

The travellers were grouped according to the reason for travel as follows: Finnish resident travelling for tourism, Finnish resident travelling for work or education, expatriate, visiting friends and relatives, recently arrived immigrant (immigrant having not visited the country of origin or other malaria-endemic areas after immigration) and foreign visitor.

Countries of birth and countries where the infection was contracted were grouped as follows: sub-Saharan Africa, Southeast Asia, Central Asia and the Indian subcontinent, South and Central America and the Caribbean, North Africa, West Asia, Northeast Asia and Oceania, as modified from GeoSentinel [24]. In cases where a number of countries had been visited, the most probable country of contracting the infection was determined individually, looking at travel history and incidence of malaria in the regions visited.

Chemoprophylaxis was considered appropriate if it followed the national guidelines valid at the time of travel: mefloquine or atovaquone/proguanil or doxycycline for regions where *P. falciparum* malaria is chloroquine-resistant, and atovaquone/proguanil or doxycycline for regions where it is mefloquine-resistant.

### Statistics

Differences between the various groups were tested using the chi-square test, student’s t-test or Mann-Whitney U-test, as appropriate. Statistical analyses were carried out with SPSS version 19.0 (Norusis; SPSS Inc., Chicago Illinois, USA).

## Results

### General characteristics

Data on a total of 265 imported malaria cases were recorded between 2003 and 2011 both by the reference laboratory and the NIDR (range, 21-40/year; average annual incidence rate, 0.5/100,000 population); the individual cases were checked and found to match in both sets of data. For the present study, detailed information about the patients was gathered solely from questionnaires in 36%, from questionnaires and original patient records in 48%, and, in addition to these, by contacting the clinician or the patient in 16% of the cases.

Table 1 presents the characteristics and data for all patients divided according to the malaria endemicity of their countries of birth. Of all cases, 55% were foreign-born, 45% were native Finns. Among those of foreign origin, 99% (144/146) were born in malaria-endemic regions, 86% (126/146) in sub-Saharan Africa. There were 25 (9%) under 18 years of age, 88% (22/25) of them born in a malaria-endemic regions, and 60% (15/25) recently immigrated into Finland. The patients originally coming from regions where malaria is endemic were younger (p=<0.001) and more often male (76% *vs* 64%, p=0.016) than those born in Finland or other non-endemic countries. No difference was found in the duration of travel between the two groups (median 35 *vs* 32 days, p=0.83).

**Table 1:** Patient characteristics and data by region of birth

**Characteristics of malaria cases**		**Total**	**Born in malaria-endemic area**	**Born in Finland or other non-endemic area**
**Number of travellers**		265	144 (54%)	121 (46%)
**Age in years, median (range)**		31(1-71)	29 (1-71)	37 (0-71)
**Sex (male)**		187(71%)	110 (76%)	77 (64%)
**Country of residence**	Malaria-endemic area	36(14%)	22 (15%)	14 (12%)
	Finland or other non-endemic area	229(86%)	122 (85%)	107 (88%)
**Duration of travel in days**^**1**^**, median (IQR)**^**2**^		33(16-92)	32 (23-68)	35 (14-151)
**Geographic region where malaria was contracted**	Sub-Saharan Africa	214(81%)	126 (88%)	88 (73%)
	Central Asia and Indian subcontinent	19 (7%)	7 (5%)	12 (10%)
	Southeast Asia	18 (7%)	7 (5%)	11 (9%)
	South and Central America and Caribbean	8 (3%)	1	7 (6%)
	Other (North Africa, Oceania)	6 (2%)	3 (2%)	3 (2%)
**Reason for travel**	Visiting friends and relatives	78 (29%)	76 (53%)	2 (2%) ^3^
	Finnish resident, tourism	66 (25%)	2 (1%)	64 (53%)
	Recently arrived immigrant	39 (15%)	38 (26%)^4^	1 (1%)
	Foreign visitor	28 (11%)	23 (16%)	5 (4%)
	Expatriate	28 (11%)	2 (1%)	26 (21%)
	Finnish resident, work/education	26 (10%)	3 (2%)	23 (19%)
**Received pre-travel advice**^**5**^		102/209(49%)	19/97 (20%)	83/112 (81%)
**Used appropriate chemoprophylaxis regularly**^**6**^		13 (5%)	0	13 (11%)
**Onset of symptoms after leaving malaria-endemic area in days**^**7**^**, median (IQR)**^**2**^		6 (1-15)	3 (1-13)	8 (2-19)
**Duration of symptoms before first contact with healthcare in days**^**8**^**, median (IQR)**^**2**^		3 (1-5)	3 (1-5)	2.5 (1-4)

^1^ Data missing for 32 cases, 39 recently arrived immigrants excluded.

^2^ Interquartile range.

^3^ Children of immigrant family.

^4^ Includes four adopted children.

^5^ Data missing for 17 cases; 39 recently arrived immigrants excluded.

^6^ Data missing for three cases.

^7^ Data missing for 19 cases.

^8^ Data missing for 15 cases; four had no symptoms.

**Figure 1:**
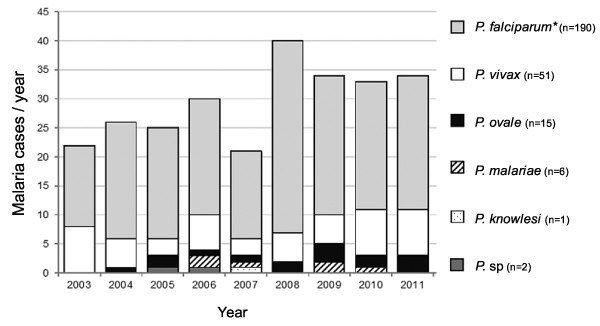
Annual number of malaria cases diagnosed in Finland by species.

The number of imported malaria cases varied between 21 and 40 per year (Figure 1). The most common species was *P. falciparum* (72%, 190/265 cases, including four double infections with other *Plasmodium* species), followed by single infections of *Plasmodium vivax* (19%, 51/265), *Plasmodium ovale* (6%, 15/265), *Plasmodium malariae* (2%, 6/265), and *Plasmodium knowlesi* (one case). In two cases the species remained unidentified. The majority of all infections (81%) and most of *P. falciparum* cases (96%) came from sub-Saharan Africa (Table 2). The countries where the disease was most commonly contracted were Nigeria (n=39 cases), Cameroon (n=23), the Gambia (n=23), Ghana (n=21), India (n=19), and Sierra Leone (n=16).

**Table 2:** Malaria cases by species and geographic region of acquiring the infection

**Geographic region**	***P. falciparum***	***P. vivax***	***P. ovale***	***P. malariae***	***P. knowlesi***	**Unidentified species**	**Total**
**Sub-Saharan Africa**	182*	10	14	6	0	2	214
**Central Asia and Indian subcontinent**	3	16	0	0	0	0	19
**Southeast Asia**	2**	15	0	0	1	0	18
**South and Central America and Caribbean**	2	6	0	0	0	0	8
**Oceania**	1	4	0	0	0	0	5
**North Africa**	0	0	1	0	0	0	1
**Total**	190	51	15	6	1	2	265

* Includes three double infections: one *P. falciparum* + *P. vivax*, one *P. falciparum* + *P. ovale* and one *P. falciparum* + *P. malariae.*

** Includes one double infection: *P. falciparum* + *P. vivax.*

### Pre-travel advice and use of prophylaxis

Leaving out 39 recently arrived immigrants, pre-travel advice was received by 49% (102/209) of the cases; 20% (19/97) of those born in endemic, and 81% (83/102) of those born in non-endemic regions (p<0.001) (Table 1). Pre-travel advice and anti-malarial drug compliance are presented in Table 3, grouped according to reason for travel.

**Table 3:** Pre-travel advice and use of chemoprophylaxis by reason for travel

**Reason of travel**	**Pre-travel advice received**^**1**^	**Use of chemoprophylaxis**^**2**^			**Chemoprophylaxis appropriate + taken regularly according to prescription**^**3**^
		**No chemoprophylaxis**	**Taken, but not regularly**	**Taken regularly according to prescription**	
**Finnish resident, tourism**	41 (66%)	32 (49%)	20 (30%)	14 (21%)	6 (9%)
**Finnish resident, work/education**	19 (76%)	10 (39%)	11 (42%)	5 (19%)	4 (15%)
**Visiting friends and relatives**	17 (24%)	61 (78%)	16 (21%)	1 (1%)	0
**Recently arrived immigrant**	0	39 (100%)	0	0	0
**Foreign visitor**	3 (12%)	25 (93%)	2 (7%)	0	0
**Expatriate**	22 (82%)	12 (44%)	11 (41%)	4 (15%)	3 (11%)
**Total**	102 (41%)	179 (68%)	60 (23%)	24 (9%)	13 (5%)

^1^Data missing for 17/265 cases.

^2^Data missing for 2/265 cases.

^3^Data missing for 3/265 cases.

Most of the cases (68%) used no chemoprophylaxis; 23% did not take their anti-malarials according to prescription (40% took the drugs irregularly, 27% discontinued the chemoprophylaxis during the trip and 33% withdrew it prematurely after returning from malaria-endemic area); 9% adhered to the dosage and frequency prescribed, but 46% of them were given an inappropriate drug. Regular use of appropriate prophylaxis was reported for 5% (13/262) of all cases: for 13 of the 121 natives of non-endemic countries, but for none of the 144 originating from malaria-endemic regions (11% *vs* 0%, p<0.001) (Table 1).

Of those with *P. falciparum* malaria, 73% (138/190) had taken no chemoprophylaxis and 27% (50/190) either took appropriate drugs but did not take them according to prescription (n=25; 44% took the drugs irregularly, 36% discontinued the prophylaxis during the trip and 20% withdrew it prematurely after returning from malaria-endemic area) or did not take appropriate drugs (n=25; 44% took them according to prescription, 20% irregularly, 8% discontinued the prophylaxis while travelling and 28% withdrew it prematurely after the trip). Notably, 36% (63/175, data missing for 15 cases) of the patients with *P. falciparum* malaria reported having received pre-travel advice. Appropriate regular chemoprophylaxis was reported by 4% (8/190) in the questionnaires. After double-checking this information, it was found that none of the patients had been fully compliant (Table 4).

**Table 4:** Use of chemoprophylaxis by malaria species

**Chemoprophylaxis**	***Plasmodium falciparum***		**Other malaria species**		**Total**
**No chemoprophylaxis**	138		41		179
**Chemoprophylaxis taken**	**Appropriate chemoprophylaxis and full compliance (rechecked)**		**Appropriate chemoprophylaxis, drugs reported to be taken conscientiously**		
	**no**	**yes**	**no**	**yes**	
**Mefloquine**	17	0	6	11	34
**Atovaquone/proguanil**	4	0	4	1	9
**Doxycycline**	4	0	2	0	6
**Chloroquine**	11	0	6	1	18
**Chloroquine + proguanil**	4	0	0	0	4
**Other**	10	0	2	0	12
**Missing information**	2		1		3
**Total**	190	0	62	13	265

As to other *Plasmodium* species, there were 12 cases infected with *P. vivax* or with *P. ovale*, who were reported to have conscientiously used adequate anti-malarials; these cases may be explained by a reactivation of dormant liver hypnozoites. One patient had contracted *P. malariae* in spite of regular prophylaxis (Table 4).

### Symptoms and patients’ delay

With respect to the time span between departure from a malaria region and onset of symptoms, those born in endemic areas differed from those born in non-endemic areas (3 days *vs* 8 days, respectively; p=0.005) (Table 1). Four patients had no symptoms. Three of them were asymptomatic pregnant immigrants examined because of anaemia [25]. One was a refugee child whose siblings were diagnosed with malaria; he only had *P. falciparum* gametocytes and was not treated. No difference was found in the duration of symptoms before initial contact with healthcare between those born or not born in malaria-endemic regions (median 3 *vs* 2.5 days, respectively, p=0.493 (Table 1).

### Treatment and outcome

The time span from the first contact with healthcare to diagnosis was one day or shorter for 78% (201/259) of patients, and from presenting at hospital to diagnosis one day or shorter for 97% (254/262). After the diagnosis, specific anti-malarial treatment was started within one day in 98% of the cases (256/261).

Most of the patients (99%, 261/265) were treated in hospital; 96% (250/261) as in-patients. The median length of hospital stays was five days (IQR 3-7, data missing for 17 cases). Before the year 2010, the period of hospitalization was significantly longer than during 2010-2011 (median 5 days *vs* 3.5 days, p=0.001).

Information about the treatment was available in 98% (261/265) of the cases. The first drug used for treatment was oral quinine for 48% of the patients, intravenous quinine for 20%, artemether/lumefantrine for 12%, intravenous artesunate for 10%, chloroquine for 5% (not used in *P. falciparum* infections), mefloquine for 4%, and atovaquone/proguanil for 1%. All those who received doxycyclin were also prescribed quinine or artesunate.

Complications were only recorded for the patients treated at the HUCH (n=131): 8% of them had complications; 3% (3/93) of those born in malaria-endemic regions and 21% (8/38) of those born in non-endemic regions (p=0.001). One patient with severe complicated *P. falciparum* malaria was resuscitated because of heart arrest, and was left with a permanent brain damage. There were no deaths.

## Discussion

This is presumably the first study to survey comprehensive nationwide data on imported malaria infections, and to double-check background information initially obtained by questionnaires. While several studies have reported malaria cases in patients taking appropriate chemoprophylaxis [13,15,17,18], compliance has proved difficult to assess, which prompts the question, whether there exists significant clinical resistance to the drugs currently used in chemoprophylaxis. In the present survey the questionnaires actually indicated that 4% of *P. falciparum* cases were contracted despite proper chemoprophylaxis, yet rechecking individual use of anti-malarials revealed lacking compliance for all of these cases.

### Potential under-reporting or under-diagnosing

Malaria is a notifiable disease in most European countries, but under-reporting of imported cases may exist [2,15]. The present study suggests that this does not apply to Finland: when NIDR information based on notifications from laboratories and clinicians was compared annually with data collected by the reference laboratory, it was found that the same individual cases have been identified in both. Therefore, the data presented here can aptly be considered to cover all imported malaria cases diagnosed. Its reliability is further confirmed by the fact that all the background information was checked and completed by the same specialist each year. Finnish residents diagnosed and treated for malaria abroad are not included in the data.

Although it appears that diagnosed cases are not being under-reported, diagnoses might still be missed. This concerns recently arrived immigrants in particular: while malaria among non-immune travellers is characterized by fever and various other symptoms, many studies report asymptomatic, persistent *P. falciparum* parasitaemia in immigrants from highly endemic regions even years after immigration [26-29]. In pregnant immigrants, the only symptom can be anaemia [25].

### General characteristics

The annual numbers of imported malaria cases increased during the study period – as in most other European countries [5] – which reflects increased travel to malaria-endemic countries [19], yet regions and risk groups remained the same. There was a peak in the number of cases in 2008 due to a cluster of Finnish travellers to the Gambia [30]. Distribution of the patients’ age and gender, malaria species, duration of travel, geographical regions where malaria was contracted, and risk groups resembled those in other reports from Europe [6-10,12-15]. Consistent with other studies [6-10,12-15,31], the most common species were *P. falciparum* and *P. vivax*. *Plasmodium falciparum* infections were mainly acquired in sub-Saharan Africa, and most of *P. vivax* infections on the Indian subcontinent and in Southeast Asia. A case of *P. knowlesi* was diagnosed in a Finnish traveller to mainland Malaysia in 2007 [32]. Notably, Asia is the tropical area most favoured by Finns; Thailand draws more Finnish tourists than the entire African continent [19]. The fact that most malaria cases are acquired in sub-Saharan Africa correlates with the risk of contracting malaria being highest in this region [1]*.*

Even if the majority of travellers to tropical areas are of Finnish origin, most of the patients with malaria were immigrants: 29% VFR and 15% recently arrived immigrants. The high proportion of VFRs has also been reported in several previous studies [6,7,9,10,12,13,15,31]. Notably, the proportion of recently immigrated individuals among malaria patients was high with regard to the relatively low total number of immigrants to Finland (5% of the citizens are born abroad [33]), indicating that clinicians should have a high level of suspicion for malaria when treating immigrants from endemic regions, especially sub-Saharan Africa.

### Pre-travel advice and use of chemoprophylaxis

Pre-travel advice was received less often by VFRs than Finnish tourists and residents travelling for work or education. None of the VFRs used appropriate chemoprophylaxis regularly. Poor adherence has been suggested to be due to lacking knowledge of malaria transmission and prevention [34]*,* believing that malaria is a minor illness, erroneous trust in livelong immunity, and the relatively high cost of prophylaxis [12,15]. All these data indicate that more effort should be focused on reaching immigrants planning to visit friends and relatives.

Of those diagnosed with *P. falciparum* malaria, none had taken appropriate chemoprophylaxis conscientiously, yet one third reported having received pre-travel advice. In Finland, all drugs used for malaria prophylaxis require prescription by a doctor (chloroquine only since 2006). Despite the fact that national guidelines are easily available, in five cases the doctor had incorrectly advised against the use of chemoprophylaxis. Moreover, of the 28 cases where inappropriate chemoprophylaxis was used, 19 had taken drugs prescribed by a doctor. This highlights the importance of further training for healthcare professionals. In nine cases inappropriate drugs had been bought over the counter at the destination. Pre-travel advice should emphasize the dangers of such drugs, as resorting to them may involve a risk of being given both inappropriate chemoprophylaxis and counterfeit drugs [35]. Media publicity caused by the malaria outbreak among Finnish travellers in the Gambia in 2008 may, at least temporarily, have increased awareness of risk and prevention of malaria among the public and medical practitioners alike. The data contained no patients who had used chloroquine for chemoprophylaxis in Africa since 2008.

In the present study 4% of those with *P. falciparum* malaria had reportedly taken appropriate chemoprophylaxis, but individual rechecking revealed that none of them had complied with the regimen prescribed. Previous reports have stated that adherence to chemoprophylaxis is difficult to assess in data based on questionnaires only [10,12,13,15]*.* The present investigation demonstrates that compliance recorded by means of questionnaires cannot be relied on, but should be rechecked in all cases of malaria. Histories given by patients who are acutely ill may not be reliable; clinicians may not have asked sufficiently detailed questions about the use of prophylaxis before, during and after the trip; the patients might not be willing to reveal their poor adherence to the instructions; or they may have misunderstood them. The last point could explain why the 17 patients who had conscientiously adhered to their chemoprophylaxis before and during the trip gave it up immediately after returning from malaria regions. Taken together, the results of the present study suggest that mefloquine, atovaquone/proguanil and doxycycline continue to be effective against chloroquine-resistant *P. falciparum* malaria, when used regularly.

### Symptoms and patients’ delay

The time span between leaving a malaria region and the onset of symptoms was longer among those born in non-endemic than those born in endemic countries. This could reflect the fact that a higher proportion of those in the first group had used chemoprophylaxis: while not preventing the disease, non-appropriate chemoprophylaxis may have delayed the onset of symptoms. There was no difference in the duration of symptoms before the first contact with healthcare between the groups; 41% of all cases presented later than three days after the onset of symptoms, consistently with a Scottish study [15]*.*

### Treatment and outcome

There was no doctors’ delay from the presentation at hospital to diagnosis or starting specific anti-malarial treatment after diagnosis. It thus seems that Finnish doctors working at hospitals are familiar with the guidelines and naturally suspect malaria in febrile returning travellers. By contrast, some improvement could be expected from those working in primary healthcare: in 22% of cases, the delay from the first contact with healthcare to diagnosis was more than one day.

Most (95%) of those with malaria were treated as in-patients; this proportion is higher than reported from other centres (16-78%) [8-10]*.* In Europe the national guidelines and practices differ from each other in this respect [5]*.* In the Finnish guidelines it is recommended that malaria patients should be treated primarily as in-patients. The median length of hospital stay was five days, which accords with other surveys [10]. Notably, the treatment guidelines were changed over the study period, which was reflected in the length of hospitalization. According to the recommendations that came into effect in 2008, complicated *P. falciparum* malaria should be treated with intravenous artesunate instead of intravenous quinine plus doxycycline, and since 2010 oral artemether/lumefantrine has been recommended for uncomplicated malaria, instead of oral quinine plus doxycycline. After the latter change, the length of hospital stays has shortened to four days in 2010, and further to three days in 2011.

Consistent with other reports [16,26,36], those born in malaria-endemic regions had fewer complications than those from non-endemic countries, which is probably due to the persistence of some degree of acquired immunity in these patients. There were no malaria-related deaths during the study period, perhaps due to the small number of patients; other studies have reported case fatality rates between 0.4–0.7% [12,13,36]*.*

## Conclusions

Compliance with anti-malarial chemoprophylaxis should always be double-checked, if the disease is suspected. The data show that mefloquine, atovaquone/proguanil and doxycycline are still effective in chemoprophylaxis against *P. falciparum* malaria, when taken conscientiously.

## Abbreviations

HUCH: Helsinki University Central Hospital; NIDR: National Infectious Disease Register; PCR: Polymerase chain reaction; THL: Terveyden ja hyvinvoinnin laitos (National Institute for Health and Welfare); VFR: Visiting friends and relatives; WHO: World Health Organization.

## Competing interests

The authors declare that they have no competing interests.

## Authors’ contributions

HS conceived of and designed the study, collected the data, participated in its analysis and interpretation and drafted the manuscript. PK took part in planning the study, carried out the data analysis and interpretation, and helped to draft the manuscript. OL contributed to the interpretation of the data and writing the manuscript. AK participated in designing the study, interpretation of the data and writing the manuscript, and provided guidance throughout the entire process. All authors read and approved the final manuscript.
